# Characterization of a ferroptosis and iron-metabolism related lncRNA signature in lung adenocarcinoma

**DOI:** 10.1186/s12935-021-02027-2

**Published:** 2021-07-03

**Authors:** Jie Yao, Xiao Chen, Xiao Liu, Rui Li, Xijia Zhou, Yiqing Qu

**Affiliations:** 1 Department of Pulmonary and Critical Care Medicine, Qilu Hospital, Cheeloo College of Medicine, Shandong University, Jinan, China; 2 Department of Respiratory Medicine, Tai’an City Central Hospital, Tai’an, China; 3 Department of Pulmonary and Critical Care Medicine, Qilu Hospital of Shandong University, Jinan, China; 4 Shandong Key Laboratory of Infectious Respiratory Diseases, Qilu Hospital of Shandong University, Jinan, China

**Keywords:** Ferroptosis, Iron metabolism, Signature, lncRNA, Immune infiltration, Lung adenocarcinoma

## Abstract

**Background:**

Long non-coding RNAs (lncRNAs) are increasingly recognized as the crucial mediators in the regulation of ferroptosis and iron metabolism. A systematic understanding of ferroptosis and iron-metabolism related lncRNAs (FIRLs) in lung adenocarcinoma (LUAD) is essential for new diagnostic and therapeutic strategies.

**Methods:**

FIRLs were obtained through Pearson correlation analysis between ferroptosis and iron-metabolism related genes and all lncRNAs. Univariate and multivariate Cox regression analysis were used to identify optimal prognostic lncRNAs. Next, a novel signature was constructed and risk score of each patient was calculated. Survival analysis and ROC analysis were performed to evaluate the predictive performance using The Cancer Genome Atlas Lung Adenocarcinoma (TCGA-LUAD) and Gene Expression Omnibus (GEO) datasets, respectively. Furthermore, multivariate Cox and stratification analysis were used to assess prognostic value of this signature in whole cohort and various subgroups. The correlation of risk signature with immune infiltration and gene mutation was also discussed. The expression of lncRNAs was verified by quantitative real-time PCR (qRT-PCR).

**Results:**

A 7-FIRLs signature including ARHGEF26-AS1, LINC01137, C20orf197, MGC32805, TMPO-AS1, LINC00324, and LINC01116 was established in the present study to assess the overall survival (OS) of LUAD. The survival analysis and ROC curve indicated good predictive performance of the signature in both the TCGA training set and the GEO validation set. Multivariate Cox and stratification analysis indicated that the 7‐FIRLs signature was an independent prognostic factor for OS. Nomogram exhibited robust validity in prognostic prediction. Differences in immune cells, immune functions and gene mutation were also found between high-risk and low-risk groups.

**Conclusions:**

This risk signature based on the FIRLs may be promising for the clinical prediction of prognosis and immunotherapeutic responses in LUAD patients.

**Supplementary Information:**

The online version contains supplementary material available at 10.1186/s12935-021-02027-2.

## Background

Lung cancer, an extremely heterogeneous disease, caused more deaths in 2017 than breast, prostate, colorectal, and brain cancers combined [29]. LUAD is one of the important sub-types of lung cancer with an increasing incidence [28]. Despite great efforts having been made in developing novel treatments but still received a poor prognosis with 5-year survival rates vary from 4% to 17% [13]. Patients with histologically similar tumors may have different outcomes due to molecular differences. Therefore, there is an urgent need to find new sensitive biomarkers for predicting survival of LUAD patients. Compared with a single biomarker, integrating multiple biomarkers into a signature would greatly improve prognostic prediction.

Iron is an essential trace element for human body. Its deficiency or excess can influence many biological processes [23]. Cancer cells exhibit an enhanced dependence on iron for growth and are dramatically more susceptible to iron depletion than non-cancer cells [21]. However, highly increased iron concentrations result in cell death through membrane lipid peroxidation, termed ferroptosis [12, 31]. Ferroptosis is an iron-dependent pathway of cell death that was discovered in recent years [18, 19]. The induction of cell death is known to be an viable approach for cancer therapy. Ferroptosis has also been identified as a potential prevention or therapeutic strategies to trigger cancer cell death, especially for malignancies that are resistant to traditional treatments [20]. Some studies have noticed the potential function of ferroptosis and iron metabolism in lung cancer development and suppression, but the detailed regulators remain unclear. Meanwhile, lncRNAs are defined as non-protein-coding transcripts larger than 200 nucleotides to distinguish them from small noncoding RNAs [16]. LncRNAs are participated in various biological purposes, such as immune, metabolism, infection, and so on. LncRNAs have been shown to function as master regulators in various disease processes including cancer [11]. Remarkably, it has been found that lncRNAs are the crucial mediators in the regulation of ferroptosis and iron metabolism in cancer [33]. For example, LINC00336, as an endogenous sponge of microRNA 6852, regulates ferroptosis in lung cancer cells [30]. In human leukemia, overexpression of LINC00618 increased the concentrations of intracellular iron and promoted ferroptosis [32]. Only a small number of lncRNAs have been functionally well-characterized, the clinical significance of most lncRNAs, especially FIRLs, has not been investigated clearly. Therefore, it is valuable to identify key lncRNAs closely related to ferroptosis and iron metabolism with prognosis significance in LUAD.

This is a systematic investigation of the underlying prognostic significance of FIRLs in LUAD. Prognostic FIRLs were selected using univariate Cox analysis based on TCGA database. Then, a 7‐FIRLs signature was constituted by multivariate COX regression and GEO dataset was applied for external validation. Multivariate Cox and stratification analysis verified that the independence and universal adaption of the 7‐FIRLs signature. Considering the potential role of the FIRLs in the interaction between immune infiltrating and tumor mutation burden (TMB), their relationship was further explored. In conclusion, the signature played an important role in LUAD and was potential prognostic biomarker.

## Materials and methods

### Patient data sets

The data collected in this study were from TCGA-LUAD^1^ and GEO^2^ (GSE3141, GSE37745) datasets. The detailed gene expression information, OS events and time were obtained from above three datasets, whereas clinical features data were available from TCGA (n = 477) as training set (Table1). Only LUAD patients with clear survival time and survival status were included in the study. And patients in TCGA-LUAD whose OS less than 30 days were removed in order to improve the accuracy of study. The GSE3141 (n = 58) and GSE37745 (N = 106) data sets were merged into an independent validation set. Besides, the somatic mutation data of patients with LUAD were downloaded with a mutation annotation format (MAF) file from TCGA. The “maftools” package [22] in R software was used to visualize the mutation data and calculate the TMB of LUAD patients.

**Table 1:** Clinical features of lung adenocarcinoma (LUAD) patients in TCGA database

Feature	N (477)	%
Age (years)		
≦ 65	247	51.8
> 65	230	48.2
Vital status		
Alive	320	67.1
Dead	157	32.9
Gender		
Female	257	53.9
Male	220	46.1
TNM stage		
Stage I	253	53.0
Stage II	113	23.7
Stage III	78	16.4
Stage IV	25	5.2
Unknown	8	1.7
T stage		
T_1_	159	33.3
T_2_	254	53.2
T_3_	43	9.0
T_4_	18	3.8
Unknown	3	0.6
N stage		
N_0_	307	64.4
N_1_	90	18.9
N_2_	67	14.0
N_3_	2	0.4
Unknown	11	2.3
M stage		
M_0_	313	65.6
M_1_	24	5.0
Unknown	140	29.4

### Identification of FIRLs in LUAD

According to the lncRNAs annotation file acquired from the GENCODE^3^ [8]. 14,142 lncRNAs were identified in the TCGA, and 1632 lncRNAs were identified in the GSE37745 for gene screening. Ferroptosis related genes were obtained from three databases. 177 ferroptosis regulators (including 108 drivers, 69 suppressors) and 111 ferroptosis markers were found from FerrDb database^4^ [36]. 25 ferroptosis-related genes were obtained in the ferroptosis pathway (map04216) from the KEGG PATHWAY Database.^5^ 40 ferroptosis-related genes were extracted in gene sets “M39768: Ferroptosis” from the Molecular Signatures Database (MSigDB).^6^ Iron metabolism related genes were also obtained in gene sets “M962: Iron uptake and transport” and “M15748: Iron ion homeostasis” from MSigDB. Finally, the 296 ferroptosis and iron metabolism related genes were included for subsequent research by integrating intersection genes and eliminating unrelated genes (see Additional file 5: Table S1). Pearson correlation analysis was performed between the lncRNAs and 296 ferroptosis and iron metabolism related genes (with the |Correlation Coefficient| > 0.3 and *p* < 0.001). Then FIRLs were obtained in TCGA and GSE37745 respectively. Venn analysis was used to screen intersection FIRLs from two datasets above for further analysis.

### Construction of FIRLs prognostic signature for LUAD

According to the clinical data of LUAD cases in the TCGA, univariate Cox proportional hazards regression analysis was applied to screen prognostic lncRNAs related to OS. Those lncRNAs with P value < 0.01 were selected for multivariate Cox regression analysis to identify optimal prognostic lncRNAs. A risk signature was then established on the basis of the expression levels as well as the risk coefficients of optimal prognostic lncRNAs. Based on the following formula, the risk score for each patient was calculated.

$$ {\text{Risk}}\;{\text{score}} =   {\text{Exp}}_{{{\text{lncRNA}}1}}  \times \upbeta _{{{\text{lncRNA}}1}}  + {\text{Exp}}_{{{\text{lncRNA}}2}}  \times \upbeta _{{{\text{lncRNA}}2}}  +  \cdots  + {\text{Exp}}_{{{\text{lncRNAn}}}}  \times \upbeta _{{{\text{lncRNAn}}}} , $$

lncRNA_n_ is the nth selected lncRNAs.

### Evaluation of the prognostic signature containing 7 FIRLs

LUAD patients in TCGA were divided into high-risk group and low-risk group by using the corresponding median risk score as the cutoff point. Kaplan–Meier survival analysis was performed to estimate the survival difference between the two groups by using the “survival” and “survminer” R packages. ROC curve was performed and area under the curve (AUC) at different time points were calculated to assess the diagnostic value of risk signature. Due to the few samples in GSE37745, the GSE3141 (n = 58) and GSE37745 (N = 106) data sets were merged as an independent validation set to assess the prognostic performance of the signature. The same prognostic formula and cutoff point (median risk score in TCGA) were used to calculate the risk score of each included patient and divided into high/low risk group. Next, Kaplan–Meier survival analysis and ROC curve were also performed in validation set. Besides, principal component analysis (PCA) and t-SNE analysis were performed using “Rtsne” and “ggplot2” packages to exam the clustering ability of risk signature.

Univariate and multivariate Cox regression analysis evaluated whether the risk score was independent of other clinicopathological parameters, including age, gender, TNM stage, T stage and N stage (M stage had a large number of uncertain values, which were not included in the study). The hazard ratios (HR) and 95% confidence intervals (CI) were estimated. Then a nomogram was formulated by employing “rms” R packages. All independent prognostic factors identified by multivariate Cox regression analysis were included in the construction of a prognostic nomogram to investigate the probability of 1-, 3-, and 5-OS of LUAD. Calibration curves of the nomogram were plotted to estimate the accuracy of actual observed survival rates with the predicted survival probability. But beyond all that, stratification analysis was also performed to detect the prognostic value of risk signature in different subgroups. All statistical analysis were conducted using R software and Bioconductor. The significance was defined as P value being less than 0.05.

### Functional enrichment analysis and immune infiltration level analysis

In order to investigate the biological roles of the seven lncRNAs in LUAD, the mRNAs that highly related with these above lncRNAs were identified. A co-expression network of the seven lncRNAs-mRNAs was established and visualized using Sankey diagram. The correlation coefficient threshold was set to > 0.3 or < − 0.3, and the corresponding P value < 0.01 was considered statistically significant. Functional enrichment analysis were conducted including gene ontology (GO) and Kyoto encyclopedia of genes and genomes (KEGG) pathway. The pathways with P value < 0.05 were considered as significantly enriched. The relationship between 7 lncRNAs and ferroptosis was verified by the correlation expression with four most common ferroptosis-related mRNAs through GEPIA.^7^

At the same time, the TIMER [18, 19], CIBERSORT [6, 25], QUANTISEQ [10], Microenvironment Cell Populations-counter (MCP-counter) [5], XCELL [1], and Estimating the Proportion of Immune and Cancer cells (EPIC) [27] algorithms were used to estimate the abundances of immune cells between the high-risk and low-risk groups based on FIRLs signature. In addition, ssGSEA was used to quantify the immune cells and pathways between two groups using the “gsva” package. Given the important roles of immune infiltration cells in the tumour microenvironment, the relationship between the signature and single lncRNA contained in it and immune infiltration cells were also analyzed through TIMER and CIBERSORT algorithms. Pearson correlation coefficient and P value were calculated. The expression level of immune checkpoint related genes may be connected with treatment responses of immune checkpoint inhibitors. The relationship between risk score and immune checkpoint was explored by testing the difference of gene expression level in high-risk and low-risk groups.

### Cell culture and qRT-PCR

Human LUAD cells (A549 and H1299) and normal bronchial epithelial cell (16HBE) were purchased from Cell Bank, Institute of Life Sciences, Chinese Academy of Sciences Cell Bank (Shanghai, China) and confirmed by short tandem repeat (STR) profiling. The 16HBE and H1299 cells were cultured in RPMI 1640 medium (Gibco, Invitrogen, Carlsbad, CA), and A549 cells were cultured in DMEM medium (Gibco) with 10% fetal bovine serum (FBS) under a humidified atmosphere of 37 °C and 5% of CO_2_. Total cellular RNA was extracted using TRIzol reagent (Invitrogen, Carlsbad, CA, USA) and quantified by NanoDrop Lite spectrophotometer (Thermo Scientific). The total RNA underwent reverse transcription using the PrimeScript™ RT Reagent Kit (Takara, Dalian, Liaoning, China) for cDNA synthesis according to the manufacturer’s instruction. The relative lncRNA expression levels were determined by qRT-PCR in triplicate on the Applied Biosystems StepOnePlus Real-Time PCR System (Termo Fisher Scientific) using the TB Green™ Premix Ex Taq™ II (TaKaRa). All program steps of qRT-PCR are performed in accordance with the instructions provided by the manufacture. Melting curves were generated at the end of amplification to ensure the specificity of the PCR products. GAPDH was used as an internal control. The relative expression of each lnRNA was calculated by 2^−△△Ct^ method. Multiple primers of C20orf197 were designed, and none of them had specific melting curves, so only the other six lncRNAs were conducted qRT-PCR analysis. Primers sequences are listed in Additional file 6: Table S2.

## Results

### Identification of shared FIRLs from TCGA and GEO databases

**Fig. 1:**
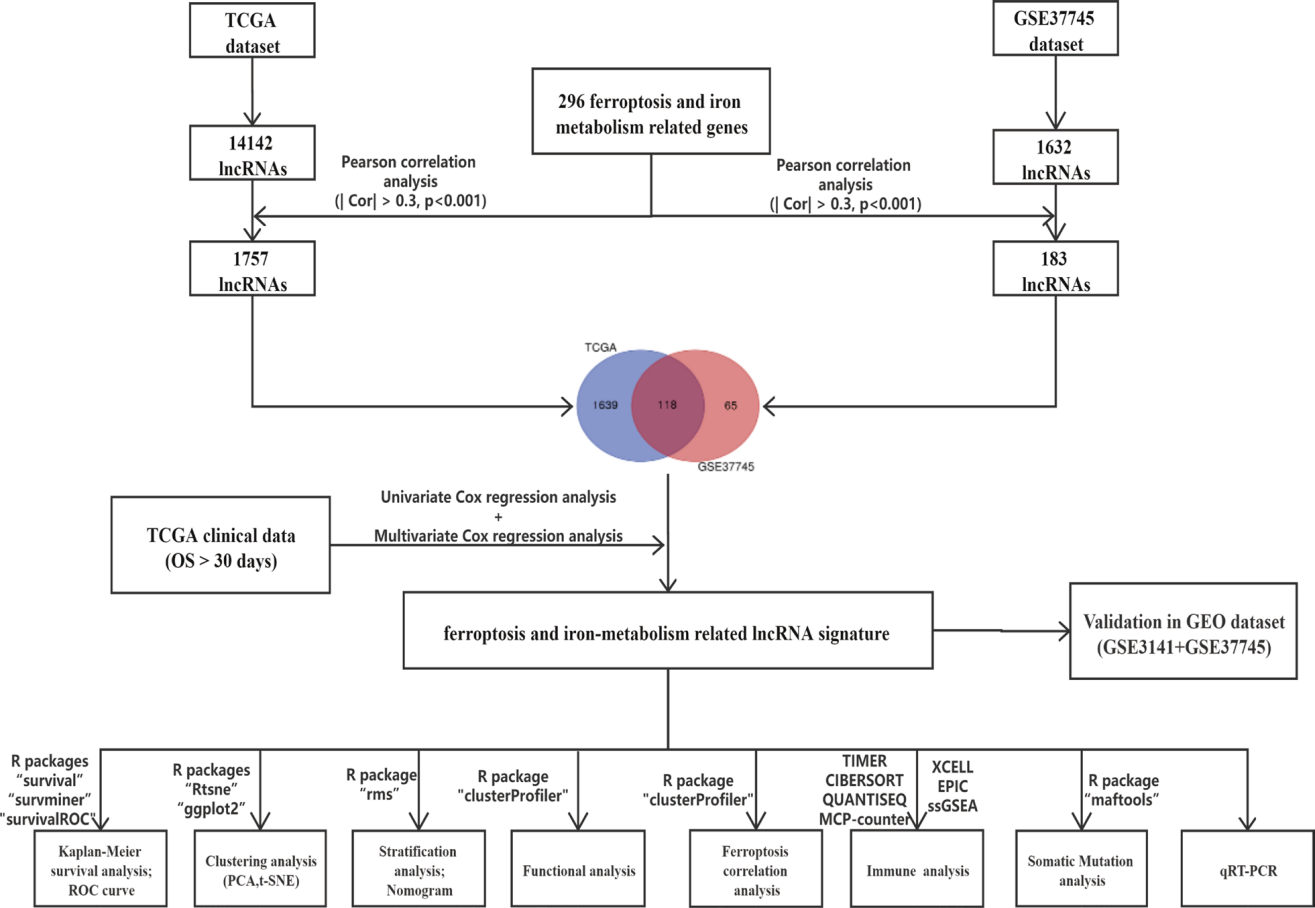
Flow chart of the study

This study was conducted according to the flow chart shown in Fig. 1. First, 14,142 lncRNAs were identified in the TCGA-LUAD and 1632 lncRNAs were identified in the GSE37745 according to the lncRNA annotation file. Then these lncRNAs were subjected to Pearson correlation analysis with 296 ferroptosis and iron metabolism related genes (Additional file 1: Figure S1A, B; |Correlation Coefficient| > 0.3 and p < 0.001). TCGA-LUAD and GSE37745 obtained 1757 and 183 FIRLs, respectively. The intersection of two datasets yielded 118 FIRLs (Additional file 1: Figure S1C; Additional file 7: Table S3).

### Derivation of FIRLs signature for OS prediction

477 LUAD patients from TCGA were chosen to explore the association between the expression of 118 lncRNAs and survival. Among 118 lncRNAs, 16 lncRNAs were significantly associated with the survival of LUAD (*P* < 0.01) by univariate Cox proportional hazards regression analysis. Then multivariate Cox proportional hazards regression analysis was performed to pick out the optimal prognostic lncRNAs with nonzero coefficients. Finally,7 lncRNAs constituted the optimal prognostic risk signature of FIRLs (Table 2), including 3 risky lncRNAs (HR > 1) and 4 protective lncRNAs (HR < 1). By combination of lncRNAs expression levels weighted by the corresponding regression coefficients, the risk score of LUAD patients was calculated as follows: Risk score = (− 0.310 × ARHGEF26-AS1) + (0.364 × LINC01137) + (− 0.270 × C20orf197) + (− 0.263 × MGC32805) + (0.331 × TMPO-AS1) + (− 0.404 × LINC00324) + (0.222 × LINC01116).

**Table 2:** The optimal prognostic risk signature of 7 lncRNAs by multivariate Cox regression analysis

LncRNA	Coef.	HR	HR.95L	HR.95H	P-value
ARHGEF26-AS1	− 0.310	0.734	0.515	1.046	0.087
C20orf197	− 0.270	0.764	0.603	0.967	0.025
MGC32805	− 0.263	0.769	0.578	1.022	0.070
LINC00324	− 0.404	0.668	0.464	0.961	0.030
LINC01116	0.222	1.249	1.039	1.501	0.018
LINC01137	0.364	1.438	1.157	1.788	0.001
TMPO-AS1	0.331	1.393	1.005	1.931	0.047

### Validation of FIRLs signature

**Fig. 2:**
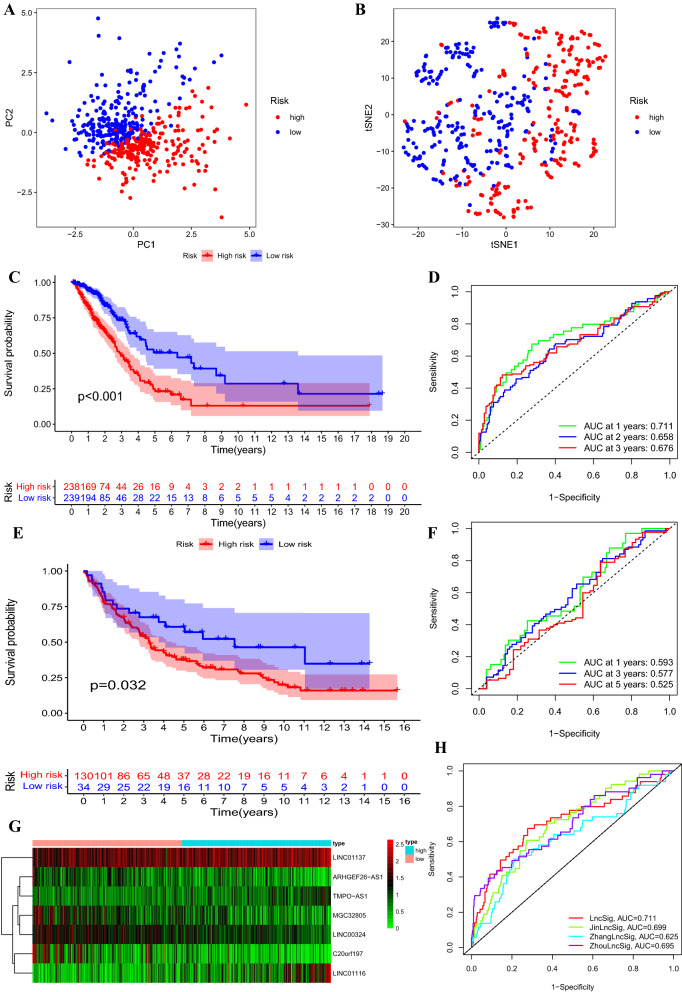
The prognostic value of the risk signature including 7 FIRLs in training set and validation set. **A**, **B** PCA (**A**) and t-SNE (**B**) analysis between high-risk and low-risk groups in training set. **C**, **D** Kaplan–Meier survival analysis (**C**) and AUC of ROC at 1-, 2- and 3-y OS (**D**) in training set. **E**, **F** Kaplan–Meier survival analysis (**E**) and AUC of ROC at 1-, 2- and 3-y OS **(F)** in validation set. **G** lncRNA expression patterns for patients in high/low risk groups based on the 7-FIRLs prognostic signature. **H** Comparison of OS prediction for different prognostic signatures. *FIRLs* ferroptosis and iron-metabolism related lncRNAs, *AUC* area under the curve, *OS* overall survival

The risk scores of all LUAD patients were obtained based on the above calculation formula. LUAD patients in TCGA were classified into high-risk group (n = 238) and low-risk group (n = 239) on the basis of median risk score. The classification ability of the risk signature was confirmed by PCA and t-SNE analysis (Fig. 2A, B). Kaplan–Meier survival analysis showed that the high-risk group exhibited a significantly shorter OS than the low-risk group (training set: *P* < 0.001, Fig. 2C; validation set: P = 0.032, Fig. 2E), indicating that the risk signature of the 7 FIRLs has prognostic value. We next assessed the predictive sensitivity and specificity of the risk signature by ROC curves. The AUC at 1, 2, and 3 years reached 0.711, 0.658, 0.676, and 0.593, 0.577, 0.525 for training set and validation set respectively (Fig. 2D, F). The expression patterns of seven lncRNAs is shown in Fig. 2G. As expected, three risky lncRNAs was highly expressed in the high-risk group and the remaining four protective lncRNAs were up-regulated in the low-risk group.

We compared the performance for OS prediction of 7-FIRLs signature (hereinafter referred to as LncSig) and other published prognostic signatures [15, 34, 35]: the lncRNAs signature derived from Zhang’s study (ZhangLncSig), Zhou’s study (ZhouLncSig) and Jin’s study (JinLncSig). Utilizing the same TCGA patient cohort, risk scores of each signature were calculated based on normalized expression values and coefficients provided by original articles and ROC analysis was performed. As shown in Fig. 2H, LncSig achieved a AUC value is 0.711, which was higher than of JinLncSig (AUC = 0.699), ZhangLncSig (AUC = 0.625) and ZhouLncSig (AUC = 0.695). These results indicated the superiority of the 7-FIRLs signature in OS prediction of patients with LUAD. Additionally, in univariate and multivariate Cox regression analysis, LncSig was both an independent and superior prognostic factor of LUAD patients. Our signature was identified to be superior or comparable to the previous defined signatures (Additional file 8: Table S4).

### Correlation of the prognostic signature of 7 FIRLs with clinicopathological features

**Fig. 3:**
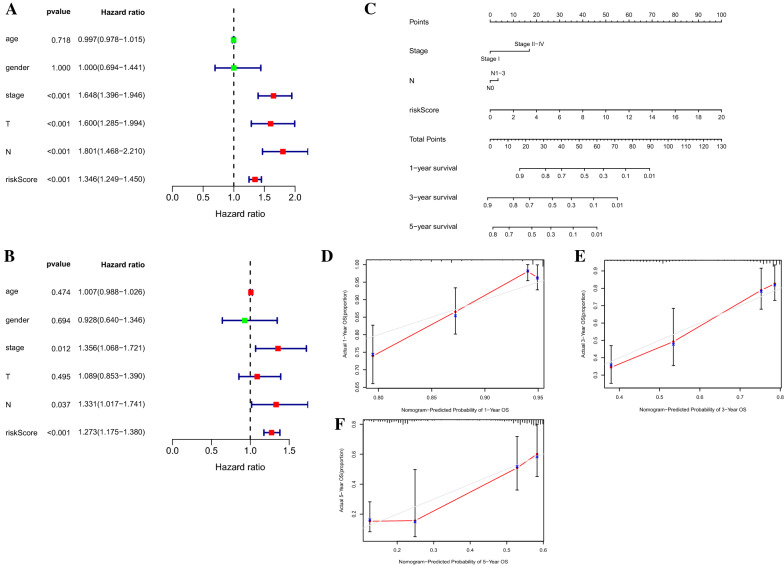
Evaluation of the risk signature including 7 FIRLs. **A** Univariate Cox regression analysis of the correlation between OS and various clinicopathological features including risk signature. **B** Multivariate Cox regression analysis revealed that the risk signature was independent prognostic factor for predicting the OS of LUAD patients. **C** The nomogram for predicting the OS of patients with LUAD at 1, 3, and 5 years. **D**, **E** Calibration curves of the nomogram for OS prediction at 1 (**D**), 3 (**F**), and 5 (**E**) years. FIRLs, ferroptosis and iron-metabolism related lncRNAs

The independence of the signature in LUAD was evaluated by univariate and multivariate Cox regression analysis. Univariate Cox regression analysis demonstrated that the risk score was associated with the OS of LUAD patients (P < 0.001; Fig. 3A). Multivariate Cox regression analysis revealed that the risk signature was independent prognostic factor for predicting the OS of LUAD patients (P < 0.001; Fig. 3B). Then a nomogram was constructed (Fig. 3C) to quantify the 1-, 3-, and 5-year survival probabilities by using independent predictive factors, including tumor stage, N stage and risk score (based on 7FIRLs signature). The calibration curves of the nomogram showed that the predicted survival rates is closed related to the actual survival rates at 1, 3 and 5 years (Fig. 3D–F).

**Fig. 4:**
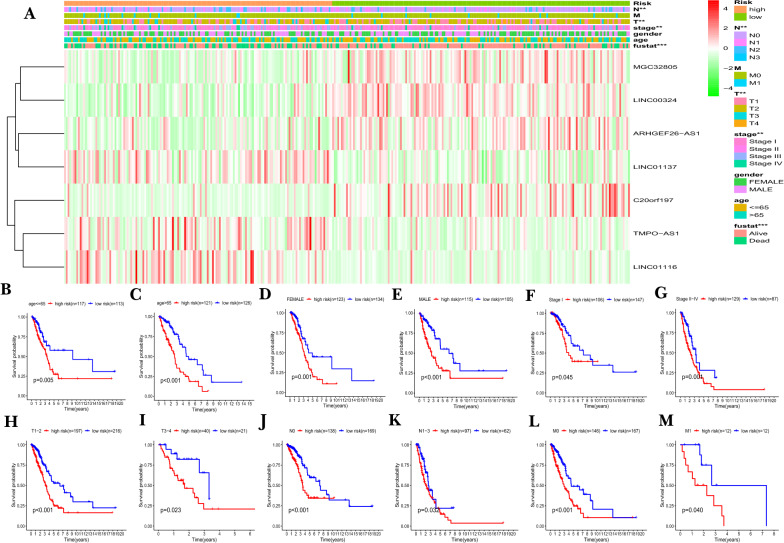
The correlation between the risk signature and different clinicopathological features. **A** 7 FIRLs expression profiles and correlation between prognostic signature and clinicopathological features. *P < 0.05; **P < 0.01; ***P < 0.001. **B**–**M** The survival differences between high- and low-risk groups stratified by clinical variables: **B**, **C** age (≤ 65 and > 65), **D**, **E** gender (female and male), **F**, **G** tumor stage (I, II and IV), **H**, **I** T stage (T_1–2_ and T_3–4_), **J**, **K** N stage (N_0_ and N_1–3_), and **L**, **M** M stage (M_0_ and M_1_). *FIRLs* ferroptosis and iron-metabolism related lncRNAs

Chi-square test was conducted to investigate whether the 7-FIRLs signature participated in the development of LUAD. The heat map (Fig. 4A) showed that there were significant differences between high- and low-risk groups in tumor stage (*P* < 0.01), N stage (*P* < 0.01), T stage (*P* < 0.01), and survival state (*P* < 0.001). Stratification analysis was further conducted using the following clinical variables: age (≤ 65 and > 65), gender (female and male), tumor stage (I, II and IV), T stage (T_1–2_ and T_3–4_), N stage (N_0_ and N_1–3_) and M stage (M_0_ and M_1_). The results indicated that the signature has prognostic significance between high and low risk patients for all subgroups. Patients in the high-risk group shown significantly poorer OS than patients in the low-risk group (Fig. 4B–M). In sum, these results testify that the 7-FIRLs risk signature exerts critical roles in determining the prognosis of LUAD patients.

### Functional enrichment analysis and immune infiltration level analysis

To explore the potential biological function of the seven FIRLs, protein-coding genes co-expressed with seven lncRNAs were screened out. |Pearson correlation coefficients | > 0.3 and p < 0.001 as the cutoff value yielded 40 protein-coding genes from the mRNA expression data of TCGA, whose expression was highly associated with all or at least one of the seven lncRNAs. A Sankey diagram was depicted to visualize the correlation of lncRNAs, mRNAs, and risk type (Additional file 2: Figure S2A). GO functional enrichment analysis revealed that the correlated mRNAs were significantly clustered in ion homeostasis and protein catabolic processes (Additional file 2: Figure S2B). The KEGG enrichment analysis showed that the correlated mRNAs were enriched in known ferroptosis, necroptosis, autophagy and cancer-related pathway (Additional file 2: Figure S2C). The correlation expression between 7 FIRLs and four most common ferroptosis-related mRNAs (FTH1, GPX4, ACSL4, PTGS2) verified the relationship between 7 lncRNAs and ferroptosis from another perspective. The results with P value < 0.05 are shown in Additional file 2: Figure S2D–L.

**Fig. 5:**
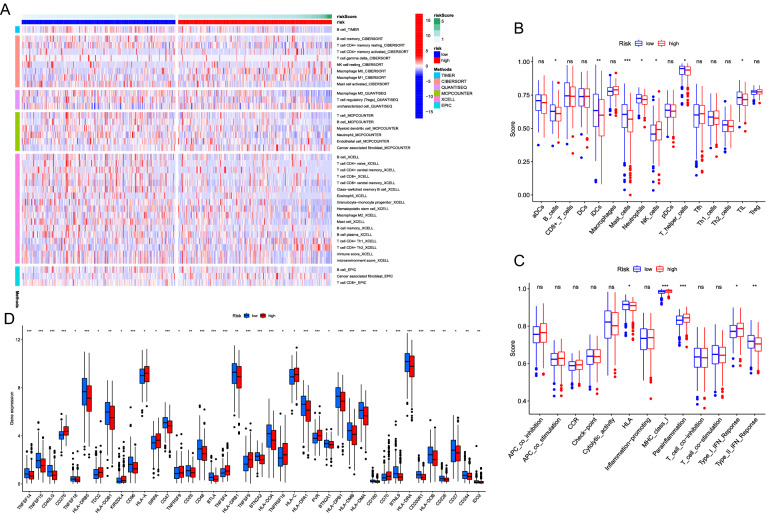
Immune infiltration analysis. **A** Heatmap for immune responses based on TIMER, CIBERSORT, QUANTISEQ, MCP-counter, XCELL, and EPIC algorithms among high and low risk groups. **B**, **C** Results for ssGSEA scores [immune cells scores (**B**) and immune functions scores (**C**)] between high and low risk groups in boxplots. **D** Expression of immune checkpoints among high and low risk groups. *ns* not significant; *P < 0.05; **P < 0.01

The heatmap of immune infiltration based on TIMER, CIBERSORT, QUANTISEQ, MCP-counter, XCELL, and EPIC algorithms is shown in Fig. 5A. Comparative analysis of immune cells and pathways confirmed the differences of HLA, MHC class I, parainflammation, type I IFN response, type II IFN response, B cell, iDCs, mast cell, neutrophils, NK cell, T helper cell and TIL between two risk groups (*P* < 0.05, Fig. 5B, C). Given the importance of checkpoint-based immunotherapy, difference was further found in the expression of immune checkpoints between two-groups (Fig. 5D). Then, scatter plots were generated using TIMER database to show the relationship between the risk score and immune cell infiltration. Results showed that the immune cell infiltration was negatively correlated with the prognosis of LUAD patients (Additional file 3: Figure S3A–F): B cells (cor = − 0.181, p = 7.595e−05), CD4 cells (cor = − 0.105, p = 0.023), CD8 cells (cor = − 0.061, p = 0.185), dendritic cells (cor = − 0.08, p = 0.082), neutrophil (cor = − 0.043, p = 0.353), macrophages (cor = − 0.112, p = 0.015). It was suggested that this prognostic signature may participate in immune response in tumor microenvironment through affecting immune cells. Moreover, CIBERSORT algorithm was applied to analysis the correlation between expression level of single lncRNA in the signature and immune cell infiltration (p < 0.001, Additional file 3: Figure S3G-N). In summary, these results indicate that the 7-FIRLs prognostic signature of LUAD was correlated with immune cell infiltration to a certain extent.

### Somatic mutations in different risk groups based on 7-FIRLs signature

**Fig. 6:**
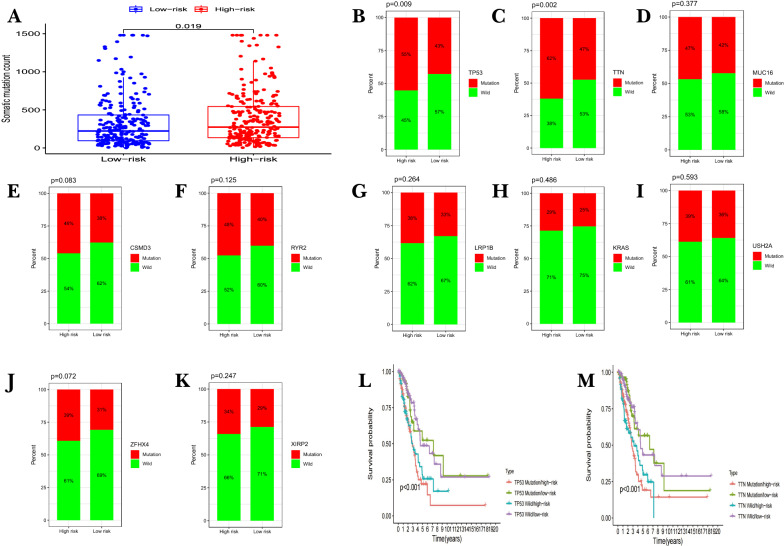
Mutation analysis between high/low risk groups. **A** Comparative analysis of mutation event between high/low risk groups. **B**–**K** Differential analysis of the top ten mutated genes between the high/low risk groups. **L**, **M** Survival analysis based on TP53 (**L**)/TTN (**M**) mutation status and low/high risk

The somatic mutation information of LUAD patients were utilized to explore the association between risk score and TMB. First, detailed mutation information of each gene was exhibited in waterfall plot, where small rectangles with different color represent different mutation types (Additional file 4: Figure S4A). Among these mutations, missense mutation was the most common type in patients with LUAD (Additional file 4: Figure S4B). Single nucleotide polymorphism (SNP) occurred more proportion than insertion (INS) or deletion (DEL), and C > A was the most common of single nucleotide variants (SNV; Additional file 4: Figure S4C, D). The number of variants per sample was shown in Additional file 4: Figure S4E. The box diagram showed the mutation type with different colors (Additional file 4: Figure S4F). Horizontal histogram revealed the top ten mutated genes with high mutation frequency (Additional file 4: Figure S4G). Then, patients in the high-risk group were found with more mutation event than patients in the low-risk group (p < 0.05; Fig. 6A). Differential analysis was performed with the top ten mutated genes between the high/low risk group (Fig. 6B–K). The results showed that the TP53 mutation and TTN mutation had statistically significant differences between two groups. Then LUAD patients were divided into four groups for survival analysis based on TTN/TP53 mutation status and risk scores. Significant difference was found in OS between the four subgroups, and patients in the TP53 mutation/high-risk group suffered shorter survival time than those in the TP53 wild/low-risk group (Fig. 6L). The same results were found for TTN group (Fig. 6M). Results also showed that the higher risk score was associated with lower survival probability in TTN/TP53 mutation subgroups, suggesting that the 7 FIRLs signature acted as a risk factor for patients carrying *TTN/TP53* mutation.

### Validation the expression of lncRNAs in LUAD cells

**Fig. 7:**
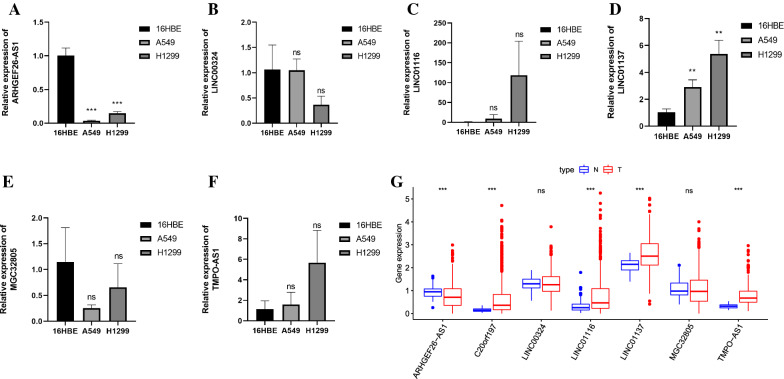
Validation the expression of FIRLs. **A** ARHGEF26-AS1, **B** LINC00324, **C** LINC01116, **D** LINC01137, **E** MGC32805, **F** TMPO-AS1 expression in normal (16HBE) and LUAD cell lines. *P < 0.05; **P < 0.01; ***P < 0.001. **G** Heat map of lncRNAs expression in TCGA

We applied the unpaired t test to assess the expression levels of the 7 lncRNAs in LUAD cell lines by qRT-PCR. As show in Fig. 7A–F, the expression of LINC01137 was upregulated while ARHGEF26-AS1 was downregulated in LUAD cells. Although the expression of MGC32805, TMPO-AS1, LINC00324 and LINC01116 showed no statistical difference between LUAD cells and normal bronchial epithelial cell (16HBE), their expression trend was consistent with bioinformatics analysis (Fig. 7G). The qRT-PCR data revealed that our bioinformatics analysis was accurate.

## Discussion

Recently, the next-generation sequencing has been transformative for the prognosis prediction of cancer [2, 3, 17]. In routine clinical practice, pathologic staging is a vital prognostic determinant of LUAD. However, clinical outcomes differ among patients at the same stage, indicating that the traditional staging system cannot adequately predict the prognosis of patients. Biomarkers related to tumor diagnosis and prognosis urgently need to be developed. Disturbances in iron metabolism cause excessive intracellular iron storage and may induce ferroptosis [4]. Impaired ferroptosis is implicated in various pathological conditions [7]. Due to the important role of ferroptosis and iron metabolism in cancer, its related lncRNA has also attracted a lot of attention [24].

To the best of our knowledge, this study is the first one to identify and comprehensively analyze prognostic FIRLs in LUAD. This signature based on 7 FIRLs provides a useful tool to supplement the traditional clinical prognostic factors, and guides prognostic prediction and therapeutic decisions. Additionally, we provide a FIRLs-related nomogram combining clinical factors to predict the OS of LUAD patients with an effective quantitative approach.

Immune regulation plays a crucial part in the progression of LUAD. The number and proportion of infiltrating immune cells are recognized as important factors affecting cancer progression and immunotherapy response and associated with patient prognosis. According to the tumor immunoediting hypothesis [9], less immunogenic cancer cells are selected for during tumor development in immune-competent hosts, to evade antitumor immune responses. This may result in increased immunosuppressive cells (e.g., regulatory T cells), decreased immunoreactive cells (e.g., helper T cells). Thus, we hypothesized that patients in different risk groups would have different immunotherapeutic responses. Results found that high-risk LUAD patients had higher NK cells infiltration and lower fractions of Mast cells and helper T cells than low-risk patients. The above results suggest that the poorer prognosis of high-risk patients is due to higher immunosuppression and lower immunoreactivity in the tumor microenvironment, and these differences contribute to tumor progression. Checkpoint inhibitor-based immunotherapies have improved the survival of patients of advanced malignancies [14]. Significant differences in the expression of immune checkpoints between high and low risk groups suggested the differences in the sensitivity to immunotherapies. Furthermore, findings in some cancer suggest that TMB may predict clinical response to immune checkpoint inhibitors [26]. In this study, we found that patients with LUAD in high risk group had higher TMB levels which was related to the immune effect.

However, several limitations of our study should be taken into consideration. Firstly, our study was mainly based on data from TCGA in which most patients were White or Asian. Caution must be taken when extrapolating our findings to patients from other ethnicities. Secondly, external validation of the signature in large-scale multicenter cohorts is necessary. Thirdly, further functional experiments in our laboratory will be required to verify findings and elucidate the roles of FIRLs in LUAD. In addition to its excellent performance in differentiating LUAD from normal lung, the role of the signature in differentiating normal lung, pulmonary nodules, and small cell lung cancer remains to be further elucidated.

In summary, the 7-FIRLs signature is a potential tool for predicting the OS rate of LUAD patients. Importantly, the signature might be associated with immune infiltration levels and even the TMB scores. We expect this robust signature will provide clues on biological behaviors as well as prognostic characteristics in clinical tests.

## Conclusions

By and large, we successfully constructed a strong predictive signature of ferroptosis and iron metabolism which may serve as a new biomarker and therapeutic target affecting the progression of LUAD. Meanwhile, the signature helps researchers deeply understand the correlation between ferroptosis and tumourigenesis. Furthermore, this study provides a promising avenue for future anti-tumor immunotherapy.

## Supplementary Information

**Additional file 1: Figure S1.** Identification of FIRLs. **A**, **B** The correlation networks of 296 ferroptosis and iron metabolism related genes (red) and lncRNAs (green) from TCGA (**A**) and GSE37745 (**B**). **C** Venn diagram showed the intersection FIRLs from TCGA and GSE37745. FIRLs, ferroptosis and iron-metabolism related lncRNAs.

**Additional file 2: Figure S2.** Functional enrichment analysis and ferroptosis correlation analysis. **A** A Sankey diagram was depicted to visualize the correlation of lncRNAs, mRNAs, and risk type. **B**, **C** Results for GO (**B**) and KEGG (**C**) enrichment analysis of the mRNAs related with the 7 FIRLs. “BP”: biological process, “CC”: cellular component, and “MF”: molecular function. **E**–**L** The correlation expression between 7 FIRLs and four most common ferroptosis-related mRNAs (FTH1, GPX4, ACSL4, PTGS2). *FIRLs* ferroptosis and iron metabolism related lncRNAs, *GO* gene ontology, *KEGG* Kyoto Encyclopedia of Genes and Genomes.

**Additional file 3: Figure S3.** Scatter plots showed the relationship between the prognostic signature and immune cell infiltration. **A**–**F** The relationship between risk score and immune cell infiltration. **G**–**N** The relationship between expression level of a single lncRNA in the signature and immune cell infiltration.

**Additional file 4: Figure S4.** Somatic mutation information of LUAD patients. **A** Waterfall plots represent mutation information of each gene in LUAD patients. The small rectangles with different color represent different mutation types. **B**–**D** Classification of different mutation types, in which missense mutation was the most common type, SNP occurred more proportion than INS or DEL, and C > A was the most common of SNV. **E** The number of variants per sample. **F** The box diagram showed the mutation type with different colors. **G** The top ten mutated genes in LUAD. *SNP* single nucleotide polymorphism, *INS* insertion, *DEL* deletion.

**Additional file 5: Table S1.** Ferroptosis and iron-metabolism related genes.

**Additional file 6: Table S2.** Primers sequences in qRT-PCR.

**Additional file 7: Table S3.** 118 Ferroptosis and iron-metabolism related lncRNAs.

**Additional file 8: Table S4.** The results of univariate and multivariate Cox regression analysis performed to compare prognostic value of previously published signatures with LncSig developed in this study.

## Data Availability

All data generated or analysed during this study are included in this published article and its Additional files.

## Abbreviations

LNCRNAs: Long non-coding RNAs
FIRLs: Ferroptosis and iron-metabolism related LncRNAs
LUAD: Lung adenocarcinoma
TCGA-LUAD: The Cancer Genome Atlas Lung Adenocarcinoma
GEO: Gene Expression Omnibus
qRT-PCR: Quantitative real-time PCR
OS: Overall survival
TMB: Tumor mutation burden
MAF: Mutation annotation format
MSigDB: Molecular Signatures Database
AUC: Area under the curve
PCA: Principal component analysis
HR: Hazard ratios
CI: Confidence intervals
GO: Gene ontology
KEGG: Kyoto Encyclopedia of Genes and Genomes
STR: Short tandem repeat
FBS: Fetal bovine serum
SNP: Single nucleotide polymorphism
INS: Insertion
DEL: Deletion
NGS: Next Generation Sequencing

## Footnotes

1 https://cancergenome.nih.gov/.

2 https://www.ncbi.nlm.nih.gov/geo/.

3 https://www.gencodegenes.org/.

4 http://www.zhounan.org/ferrdb/.

5 https://www.genome.jp/kegg/pathway.html.

6 https://www.gsea-msigdb.org/gsea/msigdb/.

7 http://gepia.cancer-pku.cn/.
