# Supplementary figures and images for: Characterization of a ferroptosis and iron-metabolism related lncRNA signature in lung adenocarcinoma
Source: Cancer Cell Int. 2021 Jul 3;21:340. doi: 10.1186/s12935-021-02027-2 (PMC8254945; doi:10.1186/s12935-021-02027-2)

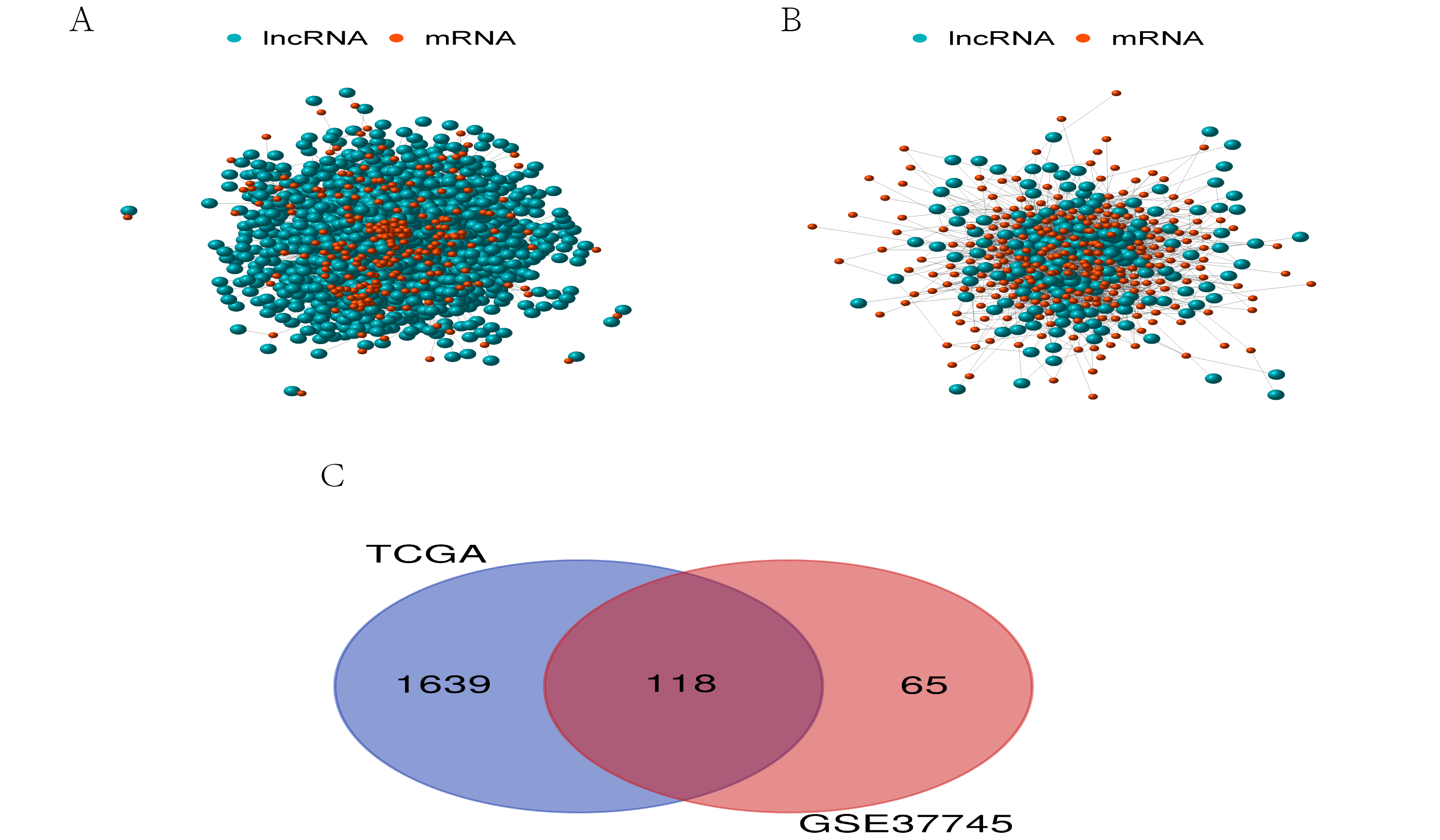

Supplement: Supplementary file 1 — Additional file 1: Figure S1. Identification of FIRLs. A, B The correlation networks of 296 ferroptosis and iron metabolism related genes (red) and lncRNAs (green) from TCGA (A) and GSE37745 (B). C Venn diagram showed the intersection FIRLs from TCGA and GSE37745. FIRLs, ferroptosis and iron-metabolism related lncRNAs. [file 12935_2021_2027_MOESM1_ESM.tif]

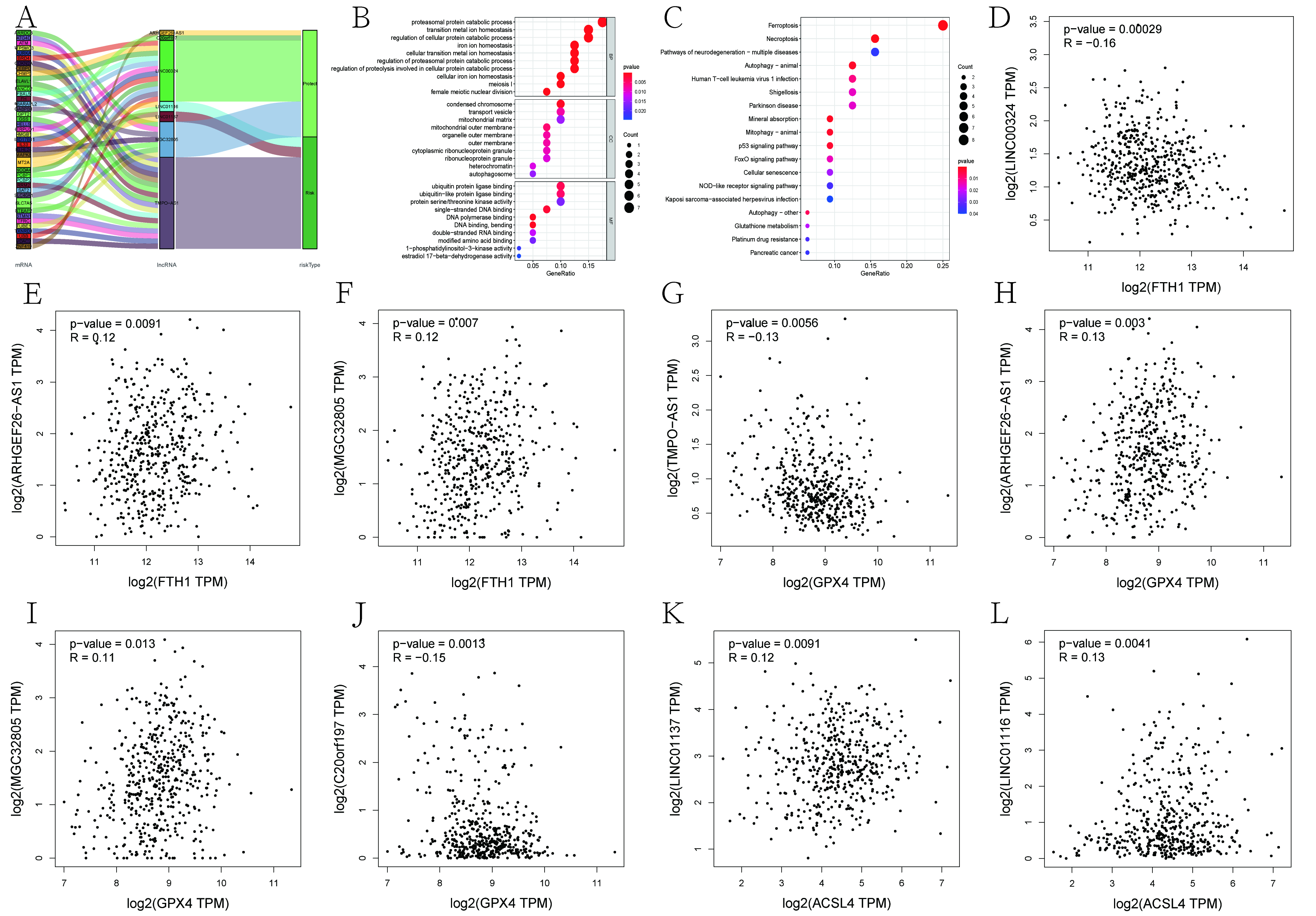

Supplement: Supplementary file 2 — Additional file 2: Figure S2. Functional enrichment analysis and ferroptosis correlation analysis. A A Sankey diagram was depicted to visualize the correlation of lncRNAs, mRNAs, and risk type. B, C Results for GO (B) and KEGG (C) enrichment analysis of the mRNAs related with the 7 FIRLs. “BP”: biological process, “CC”: cellular component, and “MF”: molecular function. E–L The correlation expression between 7 FIRLs and four most common ferroptosis-related mRNAs (FTH1, GPX4, ACSL4, PTGS2). FIRLs ferroptosis and iron metabolism related lncRNAs, GO gene ontology, KEGG Kyoto Encyclopedia of Genes and Genomes. [file 12935_2021_2027_MOESM2_ESM.tif]

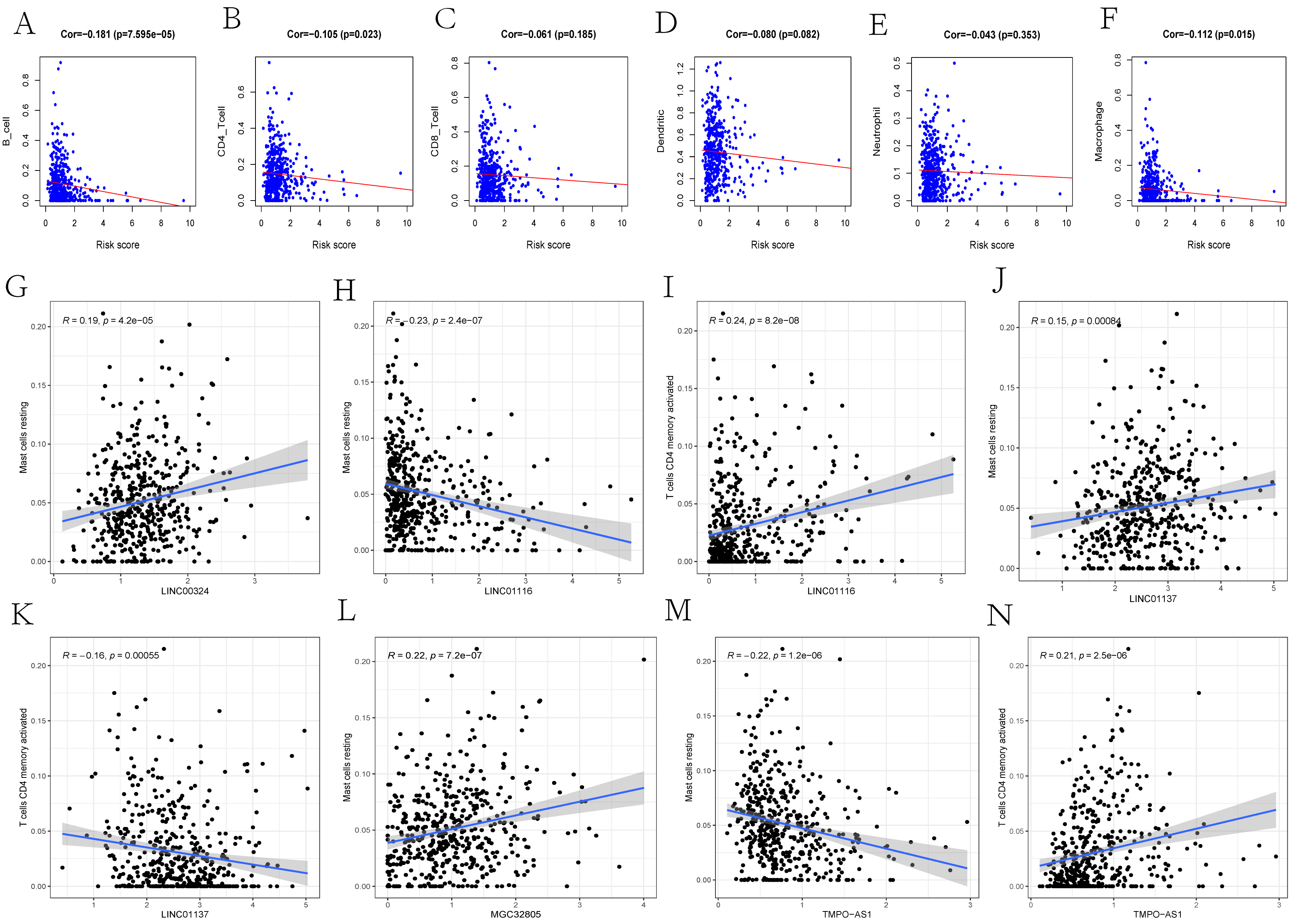

Supplement: Supplementary file 3 — Additional file 3: Figure S3. Scatter plots showed the relationship between the prognostic signature and immune cell infiltration. A–F The relationship between risk score and immune cell infiltration. G–N The relationship between expression level of a single lncRNA in the signature and immune cell infiltration. [file 12935_2021_2027_MOESM3_ESM.tif]

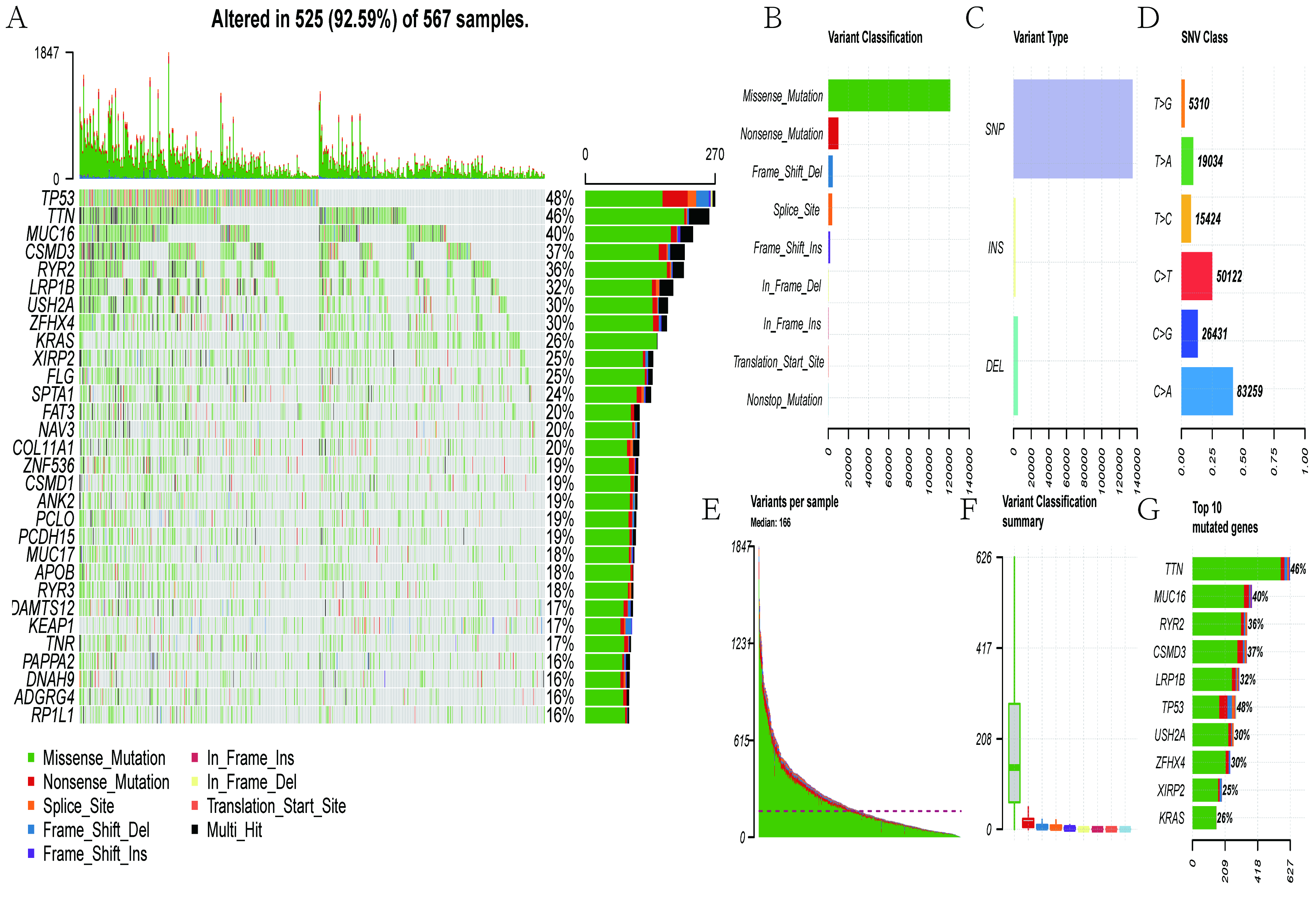

Supplement: Supplementary file 4 — Additional file 4: Figure S4. Somatic mutation information of LUAD patients. A Waterfall plots represent mutation information of each gene in LUAD patients. The small rectangles with different color represent different mutation types. B–D Classification of different mutation types, in which missense mutation was the most common type, SNP occurred more proportion than INS or DEL, and C > A was the most common of SNV. E The number of variants per sample. F The box diagram showed the mutation type with different colors. G The top ten mutated genes in LUAD. SNP single nucleotide polymorphism, INS insertion, DEL deletion. [file 12935_2021_2027_MOESM4_ESM.tif]
